# Supplementary material for: Impacts of hurricanes and disease on Diadema antillarum in shallow water reef and mangrove locations in St John, USVI
Source: PLoS One. 2024 Feb 15;19(2):e0297026. doi: 10.1371/journal.pone.0297026 (PMC10868783; doi:10.1371/journal.pone.0297026)
Supplement: Attachment — Submitted filename: Response to Reviewers - Godard et al Diadema antillarum.docx [file pone.0297026.s001.docx]

PONE-D-23-22614

Impacts of hurricanes and disease on Diadema antillarum in shallow water reef and mangrove locations in St John, USVI

PLOS ONE

**Responses to Academic Editor**

1. **Ensure that your manuscript meets PLOS ONE’s style requirements**

We have followed the specific guidelines in the PLOS ONE style templates

2. **Remove Funding Information from the Acknowledgement section and ensure that the Funding Statement is accurate.**

We have removed the funding information from the Acknowledgement section and confirm that the Funding Statement is accurate.

3. **We note that the grant information you provided in the ‘Funding Information’ and ‘Financial Disclosure’ sections do not match**.

We attempted to correct the mismatch in these two sections. The grants we received for this research were all internal institutional grants and were not available on the drop-down options of the site.

4. **We note that Figure 1 in your submission contain map/satellite images which may be copyrighted**

The map (now Figure 2) was developed by a colleague who is an artist. We have a signed form granting permission for use of the figure and the link to the uploaded image (<https://zenodo.org/record/8372679>)

5. **The raw data was not fully available**

We have uploaded the raw data which includes all transect counts for all years as well as lat-long for the sites. This original data is available (10.5281/zenodo.8395238) and we included this link in Fig 5 and 6.

**Responses to Comments from** REVIEWER #1

1. The summary statement from Reviewer 1, “*The authors aimed to explore the bioactivity response to changes in bacterial community in coral mucus and compare the difference of the bioactivities between the conditions of coral health and bleaching,”* **does not apply to our work** as we did not explore bacterial communities.
2. **Reviewer 1 was concerned about clarity, particularly in relationship to the timeline of the study**. We appreciated their recommendation to provide a visual timeline. We developed a visual timeline which is now Fig 1 in our revised manuscript. In addition, we removed the potentially confusing language related to time in several locations in the manuscript (i.e. months after or before events) as this is now captured by the figure.
3. Reviewer 1 suggested **we include photos of the species** to document morphology and evidence of disease. We have added Fig 3 (a,b,c,d) which shows: 3a clustered individuals before the hurricane; 3b remains (spines and tests) of urchins at a reef site in 2022; 3c cluster of urchins in mangrove 2022; 3d cluster of urchins at reef site adjacent to mangrove.
4. Reviewer 1 suggested we remove the map and instead have Lat/Long. We feel that the map is useful but have included lat/long in the data available (10.5281/zenodo.8395238)

**Responses to Comments from** REVIEWER #2

1. Reviewer 2 suggested we **add language to the abstract** that indicates we employed a targeted survey methodology

We did this on line 43 of abstract.

2. Reviewer 2 suggested we **remove the surface area** of the survey sites.

We did this for clarity.

3. Reviewer 2 requested that we add **more detail on the mangrove site characteristics**, including the benthos.

We did this on lines 154-159 of marked up manuscript.

4. Reviewer 2 suggested we be clearer in the results

We clarified the results in several locations including removing one anecdotal observation.

5. Reviewer 2 asked for additional information on adult to juvenile ratio and potential differences between reef and mangrove sites. We added clarifying language and have included that language 206-208 (marked manuscript) indicating the vast majority of the *D. antillarum* were adults and no difference was noted in the abundance of juveniles between coral and mangrove sites.
6. Reviewer 2 had a number of specific editorial comments. Our responses to each are noted in blue

lines 42 – Should be “…disease outbreak on populations of…”? Corrected

line 55 – 71 per square meter would be at the extreme high end of observed densities. Suggest noting that this would have been an exceptional density or perhaps using the more “average” figures that are often reported. Included the range originally reported 3 up to 71 (now lines 56-57 of marked manuscript)

lines 57 – Should be “that” not “which”. Corrected

line 67 – Females can likely produce 10 million or more eggs per spawn. See Table 1 in Pilnick et al. (2021) A novel system for intensive *Diadema antillarum* propagation as a step towards population enhancement. Eggs were collected from captive spawned animals, but it nonetheless reflects the fecundity potential of the species. Interesting – we changed language to “can produce millions of eggs per spawn” (line 69 of marked manuscript)

line 90 – Should be “that” not “which”. Corrected

lines 123-124 – Wording could be improved for clarity here. It seems like annual surveys were conducted in January from 2017 to 2020 and then in May/June in 2022 and 2023. Timeline clarified this and language was improved

line 186 – Suggest making percentage directionally explicit within the parentheses – “(96.4% reduction)”. Also, suggest being consistent with significant digits on decimals – 0.092 should be 0.09. Corrected

line 213-214 – Is this from contemporary surveys of “urchin zones” in various places or from pre 1980s die off levels? Would help the reader to make this explicit in the text. clarified

line 228 – Comma after “cover”. Corrected

lines 251-252 – This is the first time I am catching that surveys in mangrove areas may have been conducted right along the fringe within the prop roots of the trees themselves, or is this just suggesting that adjacent habitat that could have supported Diadema was lost? Do the authors think that other areas of mangrove fringe on St. John may serve as a “refugia” for D. antillarum, or is there something special about Hurricane Hole? Clarified the description of mangrove locations in the methods (now lines 154-159 in marked manuscript). There are no other robust mangrove locations outside of Hurricane Hole. Perhaps Hurricane Hole is unique in serving as a refugia certainly other mangrove locations in the Caribbean should be surveyed.

lines 262-263 – Do the blue x’s in Figure 1 represent anecdotal observations that were not included in data reported in this study? This should potentially be made explicit in the Figure 1 caption or elsewhere. Up until these lines, I had misunderstood those X’s as being the location of particularly high-density transects. Clarified the explanation in the text as well as in the figure legend (now lines 142-143 of marked manuscript).

line 277 – I would argue that this study presents an impact of the 2022 die-off of D. antillarum populations. In this sentence, perhaps add “full extent of impacts” or something along those lines. Yes – thanks for the suggestion – corrected (now line 542 of marked manuscript).
